# Differentiation between combined hepatocellular cholangiocarcinoma and hepatocellular carcinoma: comparison of diagnostic performance between ultrasomics-based model and CEUS LI-RADS v2017

**DOI:** 10.1186/s12880-022-00765-x

**Published:** 2022-03-03

**Authors:** Chao-qun Li, Xin Zheng, Huan-ling Guo, Mei-qing Cheng, Yang Huang, Xiao-yan Xie, Ming-de Lu, Ming Kuang, Wei Wang, Li-da Chen

**Affiliations:** 1 Department of Medical Ultrasonics, Institute of Diagnostic and Interventional Ultrasound, The First Affiliated Hospital of Sun Yat-Sen University, 58 Zhongshan Road 2, Guangzhou, 510080 People’s Republic of China; 2 Department of Hepatobiliary Surgery, The First Affiliated Hospital of Sun Yat-Sen University, Guangzhou, China

**Keywords:** Combined hepatocellular cholangiocarcinoma, Hepatocellular carcinoma, Ultrasomics, Liver imaging reporting and data system

## Abstract

**Background:**

The imaging findings of combined hepatocellular cholangiocarcinoma (CHC) may be similar to those of hepatocellular carcinoma (HCC). CEUS LI-RADS may not perform well in distinguishing CHC from HCC. Studies have shown that radiomics has an excellent imaging analysis ability. This study aimed to establish and confirm an ultrasomics model for differentiating CHC from HCC.

**Methods:**

Between 2004 and 2016, we retrospectively identified 53 eligible CHC patients and randomly included 106 eligible HCC patients with a ratio of HCC:CHC = 2:1, all of whom were categorized according to Contrast-Enhanced (CE) ultrasonography (US) Liver Imaging Reporting and Data System (LI-RADS) version 2017. The model based on ultrasomics features of CE US was developed in 74 HCC and 37 CHC and confirmed in 32 HCC and 16 CHC. The diagnostic performance of the LI-RADS or ultrasomics model was assessed by the area under the curve (AUC), accuracy, sensitivity and specificity.

**Results:**

In the entire and validation cohorts, 67.0% and 81.3% of HCC cases were correctly assigned to LR-5 or LR-TIV contiguous with LR-5, and 73.6% and 87.5% of CHC cases were assigned to LR-M correctly. Up to 33.0% of HCC and 26.4% of CHC were misclassified by CE US LI-RADS. A total of 90.6% of HCC as well as 87.5% of CHC correctly diagnosed by the ultrasomics model in the validation cohort. The AUC, accuracy, sensitivity of the ultrasomics model were higher though without significant difference than those of CE US LI-RADS in the validation cohort.

**Conclusion:**

The proposed ultrasomics model showed higher ability though the difference was not significantly different for differentiating CHC from HCC, which may be helpful in clinical diagnosis.

**Supplementary Information:**

The online version contains supplementary material available at 10.1186/s12880-022-00765-x.

## Background

Combined hepatocellular cholangiocarcinoma (CHC) is an extremely rare primary liver cancer, that is composed of a mixture of hepatocellular carcinoma (HCC) and cholangiocarcinoma (CCA), with more aggressive behavior and worse prognosis than HCC or CCA [1–4]. It has been reported that the clinical characteristics of CHC patients are similar to those of HCC, and 66% of CHC patients have common risk factors for HCC [5, 6]. In addition, on CT/MRI or contrast-enhanced (CE) ultrasonography (US), the imaging findings of CHC may be similar to either or both HCC and CCA [3, 7, 8].

Serum markers alpha-fetoprotein (AFP) and carbohydrate antigen 19-9 (CA19-9) were not specific for CHC, even though the combination of imaging features and tumor markers as diagnostic criteria still indicated inadequate diagnostic efficiency [6, 8, 9]. Studies have proven that the incidence of lymph node metastasis in patients with CHC is higher than that in patients with HCC, so curative surgery must be performed with systemic nodal dissection [6, 10], and localized treatments for HCC, such as transarterial chemoembolization (TACE), are not an ideal treatment for CHC in theory [11, 12]. In addition, the role of liver transplantation currently remains uncertain in this disease [9, 13]. Imaging misdiagnosis of CHC as HCC could lead to nonstandard treatments for CHC, and the correct preoperative diagnosis is still essential.

Radiomics is an imaging analysis method based on high-throughput imaging features extracted from tomographic images [14]. In recent years, radiomics based on CT/MRI has successfully shown favorable abilities in oncology research [15–17]. US is generally the preferred method for focal liver lesion (FLL) screening, and CE US has a high accuracy in the identification of same lesions as CT/MRI [18]. A study by Li Wei et al. indicated that ultrasound-based radiomics (ultrasomics) can improve the discrimination of significant liver fibrosis [19]. Peng et al. showed that ultrasomics models were helpful to distinguish the histopathological subtypes of primary liver cancer [20]. Hu et al. demonstrated that ultrasomics was a potential biomarker for microvascular invasion prediction in HCC [21]. In addition, it has been reported that ultrasomics have good performance in differentiating benign from malignant FLL and predicting tumor deposition and lymph node metastasis [22–25].

There is no report on the application of ultrasomics methods in the identification of CHC and HCC. Our research aimed to develop and validate an ultrasomics model to distinguish between HCC and CHC, and the diagnostic performance of CEUS LI-RADS version 2017 was compared with that of the ultrasomics model.

## Methods

### Patients

The study was approved by the ethical committee of our institution, and informed consent was obtained. Our retrospective study was conducted with all eligible CHC patients on the basis of the following inclusion criteria between 2004 and 2016. HCC patients who met the following inclusion criteria during this period were randomly included in our study with a ratio of HCC:CHC = 2:1. The inclusion criteria were as follows: (1) primary HCC or CHC diagnosed by histopathological examination after biopsy or surgery, (2) patients with a high risk for HCC (cirrhosis or chronic hepatitis viral infection), and (3) available CEUS examination performed 2 weeks before the operation.

The exclusion criteria were as follows: (1) unavailability of histopathological evaluation by surgery or biopsy and (2) incomplete clinicopathological data or CE US data.

**Fig. 1:**
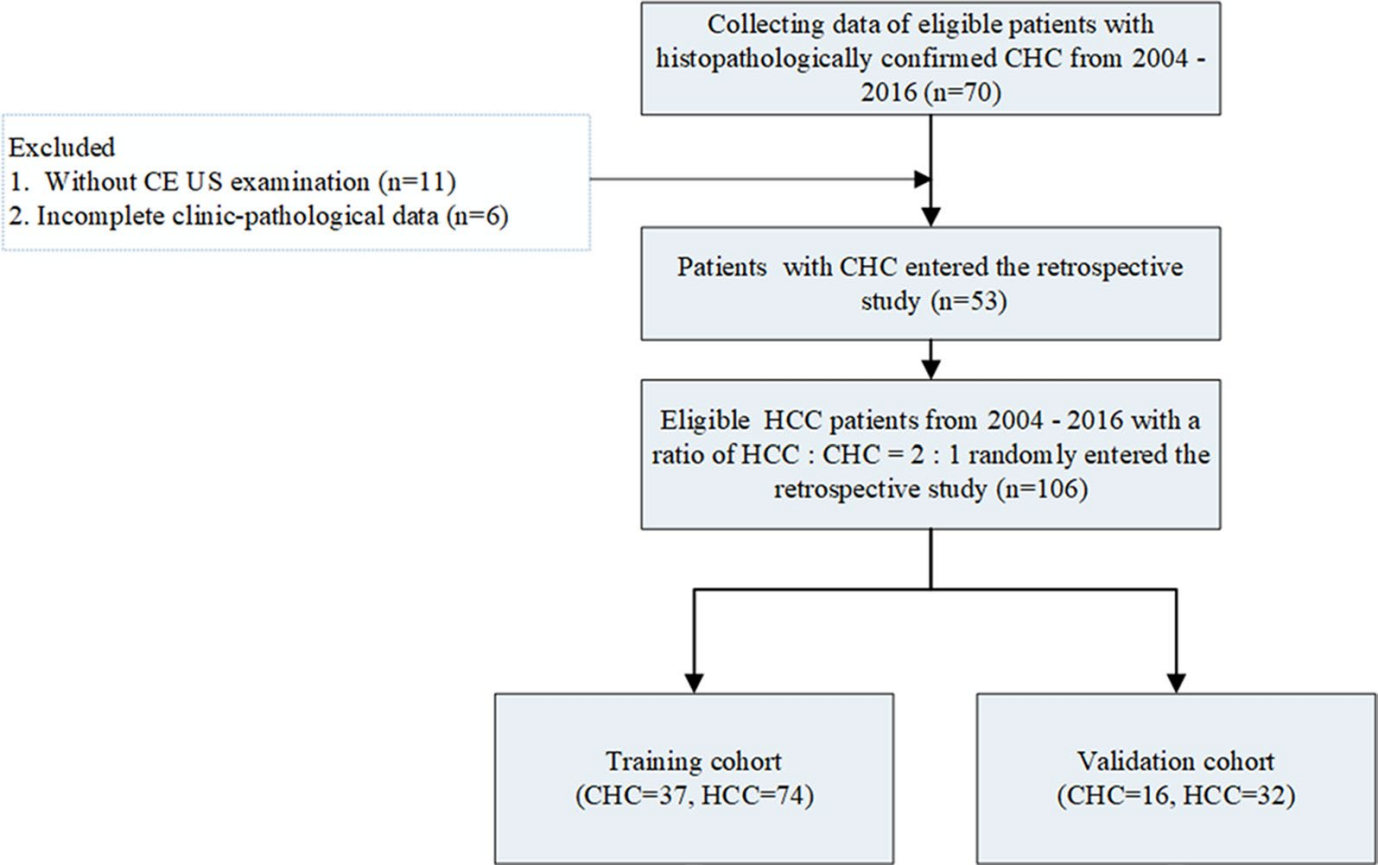
Flowchart of CHC and HCC patients’ enrollment. CHC, combined hepatocellular cholangiocarcinoma; HCC, hepatocellular carcinoma; CE, contrast enhanced; LI-RADS, liver imaging reporting and data system

The flow chart of the study population is presented in Fig. 1.

### US imaging acquisition

US studies were performed first to scan the entire liver by an experienced radiologist with the following equipment: (1) Aplio SSA-770 or Aplio 500 (Toshiba Medical Systems, Tokyo, Japan) with a 375BT convex transducer with a frequency range, of 1.9 to 6.0 MHz. (2) Acuson Sequoia 512 (Siemens Medical Solutions, Mountain View, CA, United States) with a 4V1 vector transducer with a frequency range of 1.0 to 4.0 MHz. (3) An Aixplorer Ultrasound system (SuperSonic Imagine, Aix-en-Provence, France) equipped with an SC6-1 convex probe with a frequency range of 1.0 to 6.0 MHz. If patients had multiple liver lesions, the largest lesion was regarded as the target lesion. After identifying the target lesion and storing images of recorded size, location, echo, shape, boundary, and margin, CE US examination with the same probe was performed after administration of 1.2–2.4 mL of SonoVue (Bracco Imaging, Milan, Italy) within 1–2 s into the antecubital vein followed by a 5 mL normal saline flush. The target lesion was observed continuously for at least 5 min for recording CE US features. Arterial phase hyperenhancement is described as entirely or partially (not rim-like and peripheral discontinuous globular) hyperechoic compared with the surrounding parenchyma. Washout is described as hypoechoic relative to the liver after hyperechoic or isoechoic conditions during the arterial phase. Early washout is defined as washout that occurs within 60 s after injection of the contrast agent, and marked washout is defined when a punched-out appearance (markedly hypoechoic emerging black) appears within 2 min.

### CE US LI-RADS categories

The records of the whole process of CE US examination were independently analyzed by two experienced radiologists (reader 1 and reader 2, not involved in US examinations) with more than 8 years of experience in CE US. The discussion will focus on the cases where two readers have different opinions until a final consensus is reached. All of them were blinded to the pathological and other imaging information. They were asked to classify into CE US LR-1 to LR-5 or LR-M according to CE US LI-RADS v2017. CE US LR-5 (not rim and peripheral discontinuous globular APHE with late and mild washout, meanwhile nodule size ≥ 10 mm) was defined as HCC. Then, the evaluation of the diagnostic performance of v2017 LI-RADS was conducted for HCC and CHC.

### Ultrasomics features extraction and ultrasomics models

Images of each lesion confirmed by two radiologists in consensus (including 4 images from baseline US, arterial, portal venous and late phases of CE US) were used to delineate a region of interest (ROI) around the outline of the tumor using ITK-SNAP software (version 3.6.0; www.itksnap.org). The ROI of each image included 1 cm around the lesion margin but not the portion beyond the liver parenchyma. There were 5936 features for selection in the Ultrasomics Platform (Version 2.1, Ultrasomics Artificial Intelligence X-lab, Guangzhou), including Original, Ipris, CoLIAGe, Wavelets + LBP, Shearlets etc. We selected all 5936 features extracted from a single ROI (a total of 23,744 features from each patient) using the ultrasomics platform, which mainly contains two major functions of ultrasomics feature mining and machine learning for model building. It is a kind of software for medical research that includes four essential modules of segmentation, calculation, feature selection and machine learning, and based on automatic analysis of the heterogeneity of the ROI, clinical prediction is finally achieved through the above key processes. After selecting the ratio of the training set (ratio = 0.8), median filling missing value, no oversampling and z score standardization, this module will automatically model by various combinations of feature selection methods and machine learning algorithms, and display the ROC curve of each model. The optimal model with the highest AUC was used as the final model. Finally, an ultrasomics model was developed based on features selected by Spearman rank correlation analysis, support vector machine recursive feature elimination (SVM-RFE) and machine-learning algorithms of SVM using software (details for modeling are shown in Additional file 1), and an ultrasomics score (U-score) was calculated by the ultrasomics model (U model) for each patient. The optimal cutoff value for the U model was determined using receiver operating curve (ROC) analysis. HCC was defined as a U-score of each lesion greater than the optimal cutoff value.

CHC and HCC patients finally included in this study were grouped into a training cohort and a validation cohort at a ratio of 7:3 randomly. The U model was developed in the training cohort and confirmed in the validation cohort.

### Statistical analysis

Continuous variables are expressed as the means ± standard deviations. Categorical variables are reported as numbers and percentages and were compared by the chi-square test. The optimal cutoff values for the U model were determined using ROC analysis. The diagnostic performance of the LI-RADS or U model was assessed by ROC and the area under the curve (AUC), accuracy, sensitivity and specificity with 95% confident intervals (CIs). Delong’s test was used to compare the significant differences between any two AUCs.

Statistical analysis was performed with SPSS 22.0 for Windows (Chicago, IL) and Ultrasomics-Platform (Version 2.1, Ultrasomics Artificial Intelligence X-lab, Guangzhou). *P* < 0.05 was considered statistically significant.

## Results

### Characters of patients and lesions

The final entire study cohort consisted of 159 patients (HCC = 106; CHC = 53) randomly separated into a training cohort (n = 111, HCC = 74, CHC = 37) and a validation cohort (n = 48, HCC = 32, CHC = 16). The basic characteristics of all patients and lesions are shown in Table 1. There was no significant difference in clinical and pathological characteristics between HCC and CHC (*P* > 0.05), except the levels of CA125 and CEA in CHC were significantly higher than those in HCC (*P* < 0.05) (Table 1).

**Table 1:** Basic characteristics of all patients and lesions with CE US LI-RADS classification

Characteristics	HCC (n = 106)	CHC (n = 53)	*P* value
Gender			0.778
Male	89 (84.0)	42 (79.2)	
Female	17 (16.0)	11 (20.8)	
Age (years)^a^	55.3 ± 10.9 (29—76)	53.8 ± 10.6 (25—80)	0.384
Size			0.201
< 3 cm	15 (14.2)	3 (5.7)	
3−5 cm	38 (35.8)	15 (28.3)	
> 5 cm	53 (50.0)	35 (66.0)	
Multiple lesions	13 (12.3)	13 (24.5)	0.068
AFP > 20 (μg/L)	70 (66.0)	30 (56.6)	0.324
CA199 > 35 (U/mL)	37 (34.9)	10 (18.9)	0.057
CA125 > 35 (U/mL)	8 (7.5)	16 (30.1)	< 0.001
CEA > 5 (U/mL)	7 (6.6)	13 (24.5)	0.003
LI-RADS classification			
LR-4	2	0	
LR-5	66	14	
LR-M	29	39	
LR-TIV			
With LR-5	5	0	
With LR-M	4	0	

Unless otherwise indicated, data are number of cases, with percentages in parentheses

^a^Data are means ± standard deviations, with ranges in parentheses

### LI-RADS categories and diagnostic performance

In the entire cohort, 66, 2, 29 and 9 of 106 HCC lesions were assigned to LR-5, LR-4, LR-M and LR-TIV respectively, while 25, 0, 5 and 2 of 32 HCC lesions in the validation cohort were the same (Table 1). 5 of 9 cases assigned to LR-TIV in the entire cohort were contiguous with LR-5, and the rest of LR-TIV were contiguous with LR-M. In the validation cohort, one case assigned to LR-TIV was contiguous with LR-5, and the other was contiguous with LR-M. A total of 67.0% and 81.3% of HCC in the entire and validation cohorts, respectively, were assigned to LR-5 or LR-TIV contiguous with LR-5, and most of the remaining HCC samples (31.1% and 18.8%) were assigned to LR-M or LR-TIV contiguous with LR-M. A total of 73.6% and 87.5% of CHCs in the entire and validation cohorts were assigned to LR-M, and all of the rest of the CHCs were assigned to LR-5 (Table 1). No HCC was assigned to LR-1—LR-3 and no CHC was assigned to LR-1—LR-4 and LR-TIV. The accuracy, sensitivity and specificity of CE US LI-RADS were 69.2% (95% CI 61.4–76.3), 67.0% (95% CI 57.2–75.8), 73.6% (95% CI 59.7–84.7) and 83.3% (95% CI 69.8–92.5), 81.3% (95% CI 63.6–92.8), 87.5% (95% CI 61.7–98.4) in the entire cohort and validation cohort, respectively (Table 2). The AUCs of the CE US LI-RADS (LR-5 and LR-TIV contiguous with LR-5 as a predictor of HCC) were 0.703 (95% CI 0.625–0.773) and 0.844 (95% CI 0.710–0.932) in the entire cohort and validation cohort (Table 2).

**Table 2:** Diagnostic performance of CE US LI-RADS and ultrasomics

	Entire cohort		Validation cohort		*P*
	LI-RADS	Ultrasomics	LI-RADS	Ultrasomics	
Sensitivity	67.0 (57.2−75.8)	97.2 (92.0−99.4)	81.3 (63.6−92.8)	90.6 (75.0−98.0)	0.476
Specificity	73.6 (59.7−84.7)	96.2 (87.0−99.5)	87.5 (61.7−98.4)	87.5 (61.7−98.4)	1.000
PPV	83.5 (76.0−89.0)	98.1 (93.3−99.8)	92.9 (77.9−98.0)	93.5 (78.6−99.2)	1.000
NPV	52.7 (44.8−60.4)	94.4 (84.6−98.8)	70.0 (52.6−83.1)	82.4 (56.6−96.2)	0.257
Accuracy	69.2% (61.4−76.3)	96.2% (93.3−99.2)	83.3% (69.8−92.5)	90.0% (80.9−98.2)	0.552
AUC*	0.703 (0.625−0.773)	0.981 (0.946−0.996)	0.844 (0.710−0.932)	0.895 (0.772−0.965)	0.501

Data are percentages, with 95% confidence interval in parentheses

^a^Data are area under the ROC curve, with 95% confidence interval in parentheses

### Diagnostic performance of ultrasomics model

Four kinds of important features were selected for modeling: Shearlet (10 features), CoLIAGe (4 features), Wavelet (2 features) and Gaborsp_gldp (3 features) (shown in Additional file 1). The U-score of HCC ranged from − 0.224320 to 1.531201 and from − 0.224320 to 1.513874 in the entire and validation cohorts respectively, while the U-score of non-HCC ranged from − 1.171116 to 1.001594 and from − 0.992420 to 1.001594. The optimal cutoff value for the U model obtained by using ROC analysis was − 0.0395. HCC was defined as a U-score > − 0.0395 by the U model; otherwise, CHC was defined. The U-score of lesions predicted as HCC by U model ranged from 0.004093 to 1.513874 in the validation cohorts, while the U-score of lesions predicted as non-HCC ranged from − 0.99242 to − 0.0395.

**Fig. 2:**
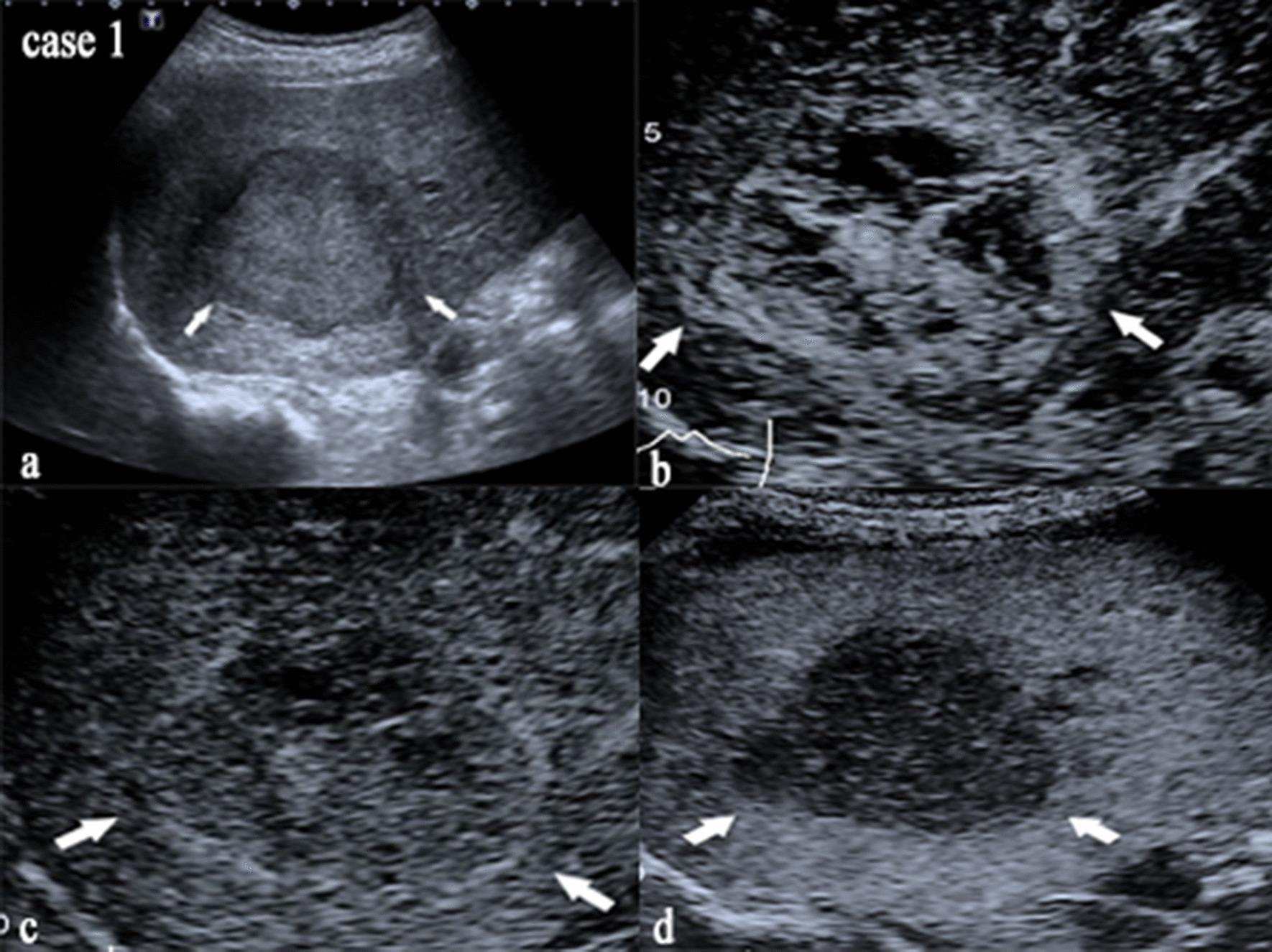
Images of US and CE US for case 1. This nodule in case 1 was diagnosed as HCC histopathologically, assigned to LR-M by CE US LI-RADS with rim arterial phase hyperenhancement and portal venous phase early washout (time of washout was 52 s), while diagnosed as HCC by U model (U-score = 0.927839594). **a** Image of nodule on US. **b**–**d** Images of nodule in arterial, portal venous and late phase on CE US

**Fig. 3:**
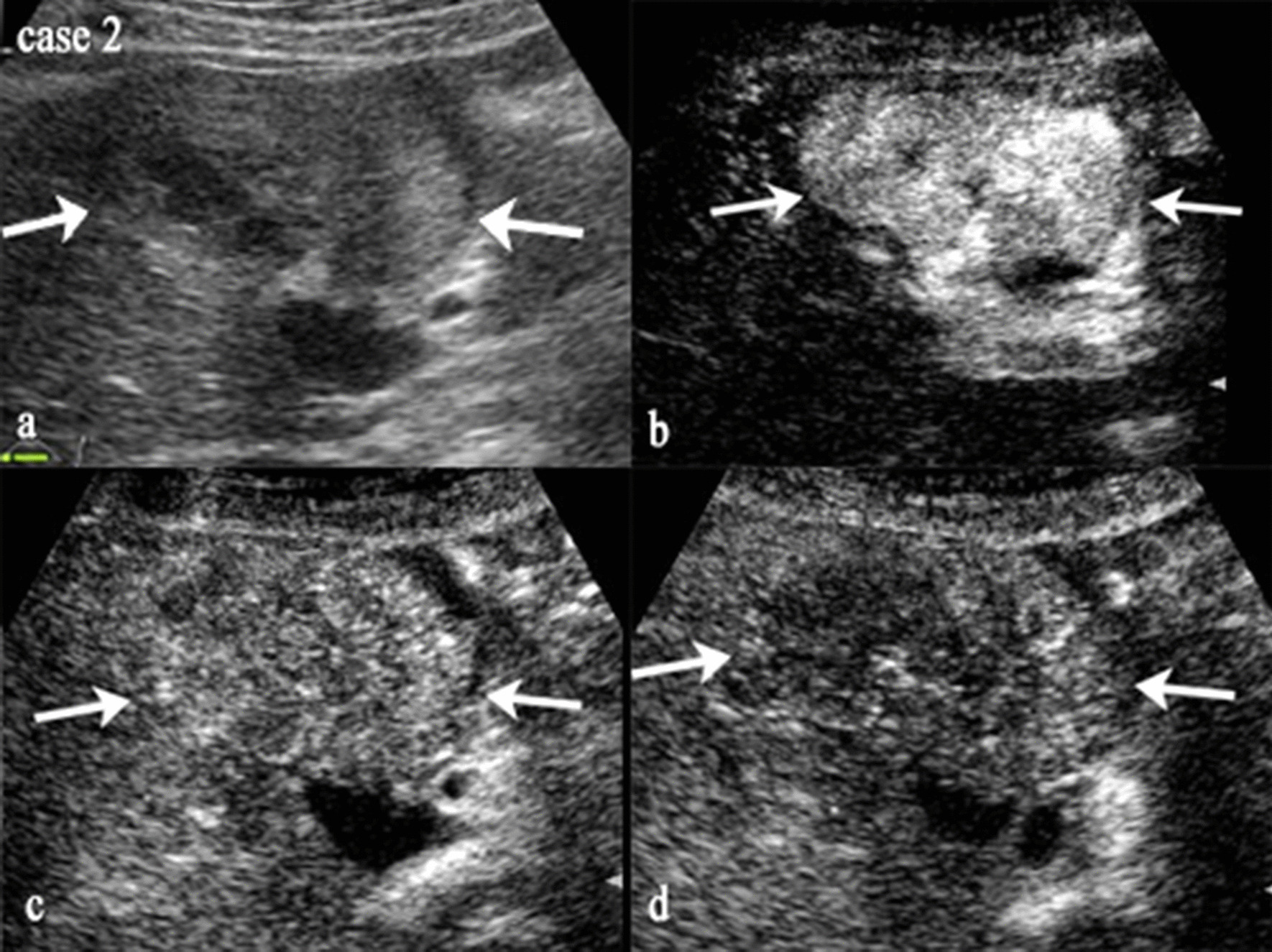
Images of US and CE US for case 2. This nodule in case 2 was diagnosed as CHC histopathologically, assigned to LR-5 by CE US LI-RADS with arterial phase hyperenhancement and portal venous phase mild, late washout (time of washout > 60 s), while diagnosed as CHC by U model (U-score = -0.236338237). **a** Image of nodule on US. **b**–**d** Images of nodule in arterial, portal venous and late phase on CE US

There were 29 of 32 (90.6%) HCC and 14 of 16 (87.5%) CHC in the validation cohort correctly diagnosed by the U model, and 103 of 106 (97.2%) HCC and 50 of 53 (94.3%) CHC in the entire cohort. Using U model, 9.8% of HCC cases assigned to LR-M/TIV in validation cohort were more accurately diagnosed than CE US LI-RADS v2017, while all CHCs misclassified as LR-5 were accurately diagnosed (Table 3). Only 3 HCC (2 assigned to LR-5, 1 assigned to LR-M) and 2 CHC (all assigned to LR-M) in the validation cohort were not confirmed by the U model, and 3 HCC (2 assigned to LR-5, 1 assigned to LR-M) and 3 CHC (all assigned to LR-M) were not confirmed in the entire cohort (Table 3). CE US images of two cases correctly diagnosed by the U model and wrongly diagnosed by CE US LI-RADS v2017 are presented in Figs. 2 and 3.

**Table 3:** CE US LI-RADS classification in cases with diagnosis of ultrasomics and pathology

	Pathology	Ultrasomics	LR-4	LR-5	LR-M	LR-TIV	
						With LR-5	With LR-M
Entire cohort (159)	HCC	HCC	2	64	28	5	4
		Non-HCC	0	2	1	0	0
	CHC	HCC	0	0	3	0	0
		Non-HCC	0	14	36	0	0
Validation cohort (48)	HCC	HCC	0	23	4	1	1
		Non-HCC	0	2	1	0	0
	CHC	HCC	0	0	2	0	0
		Non-HCC	0	2	12	0	0

The accuracy of the U model was up to 96.2% (95% CI 93.3–99.2) and 90.0% (95% CI 80.9–98.2) in the entire cohort and validation cohort respectively. The AUC of the the U model in validation cohort was increased from 0.844 to 0.895, although there was no significant difference in AUC between ultrasomics and CE US LI-RADS v2017 (*P* = 0.501) (Table 2). The sensitivity and specificity of the U model were 97.2% (95% CI 92.0–99.4), 96.2% (95% CI 87.0–99.5) and 90.6% (95% CI 75.0–98.0), 87.5% (95% CI 61.7–98.4) in the entire cohort and validation cohort, respectively. Although the sensitivity and specificity of the U model in the validation cohort were higher than those of CE US LI-RADS v2017, the differences were statistically insignificant (all *P* > 0.05) (Table 2).

### Discussion

In our study, we developed and validated an ultrasomics model for distinguishing between HCC and CHC, which proved promising differentiation ability and reliability similar to CE US LI-RADS v2017.

Previous studies on CHC were mostly focused on clinical and pathological characteristics or descriptions of imaging features, but the existing tumor biomarkers and imaging techniques are still insufficient to correctly differentiate CHC from HCC [8, 9, 26–29]. Tian et al. constructed a risk prediction model of CHC based on demographic, clinical and imaging characteristics, which presented good discrimination, but its intention was not to be a diagnostic test [30]. Two-thirds of CHC patients by enhanced CT/MR underwent imaging misdiagnosis, and the sensitivity was as low as 34% [31, 32]. Further studies proved the moderate sensitivity and specificity (61−71% and 75−85%) of using CT/MR LI-RADS to correctly categorize CHC as LR-M, and misclassification as HCC in approximately 50% of CHC [33–35]. Radiomics based on CT/MRI has shown favorable performance in the differentiation of tumors, prediction of tumor microvascular invasion, lymph node metastasis, early recurrence and prognosis [15–17]. Zhang et al. established and validated a radiomics-based model with a favorable ability for ICC differentiation of CHC [36]. A study by Liu et al. proved the promising performance of MRI radiomics features in distinguishing CHC from HCC and CCA, but CT was of limited value and the study included a small sample of only 24 CHC, 24 CCA and 38 HCC patients, which needs further validation [37]. There still existed no definite standard to distinguish patients with CHC from HCC, and there are few reports on the identification of CHC and HCC by CE US.

Based on our data, we found that there was a significant difference only in CA125 and CEA of all clinicopathologic characteristics between HCC and CHC (*P *< 0.05). There was no significant difference in AFP, which means that these tumor markers are not specific for the definitive diagnosis of CHC. Our data showed that HCC was most likely to be misclassified as LR-M and LR-TIV contiguous with LR-M (up to 31.1%), and CHC was most likely to be misclassified as LR-5 (up to 26.4%) by CEUS LI-RADS v2017. The moderate sensitivity and specificity of using LI-RADS (73.6% and 67.0% in the validation cohort) in correctly classifying CHC as LR-M in our study were similar to those in previous studies [34]. These results indicate that it is difficult to distinguish HCC from CHC by CEUS LI-RADS. Therefore, we tried to use ultrasomics features for CHC differentiation of HCC, and a comparative assessment of diagnostic performance between ultrasomics and CE US LI-RADS v2017 was conducted.

In our study, up to 26.4% of CHC cases were misdiagnosed as HCC and 31.1% of HCC cases were misdiagnosed as non-HCC malignancies by CE US LI-RADS, which means that CE US LI-RADS is imperfect to differentiate CHC from HCC. While HCC accurately diagnosed by using U model increased 9.8% and same proportion of CHC cases accurately diagnosed as LI-RADS in validation cohort, all HCC in LR-TIV contiguous with LR-M as well as 80% HCC in LR-M and all CHC in LR-5 accurately diagnosed by using U model. The AUC, sensitivity and specificity of the U model in the validation cohort were higher than those of CE US LI-RADS v2017, but there was no significant difference, which means that the ultrasomics features may have a higher ability than CE US LI-RADS v2017 for differentiating CHC from HCC, though the difference was not significantly different. Although our results, like those of the study about radiomics in differentiating CHC from CHC by Liu et al., were not perfect, the high AUC, sensitivity and specificity of the U model indicated promising discrimination ability and accuracy. We speculated that the reason for the lack of a significant difference in diagnostic performance between ultrasomics and CE US LI-RADS v2017 in the validation cohort might be the small sample size in our study. Compared with LI-RADS, the U model gives differentiation results between CHC and HCC more directly, while other non-HCC malignancies will also be classified as LR-M.

Patients with CHC that are difficult to distinguish from HCC by this model may need to consider preoperative biopsy or intraoperative pathological diagnosis for curative surgery. However, biopsy also has limitations for CHC patients. Insufficient specimens by biopsy may lead to misdiagnosis due to the difficulty of obtaining both HCC and CCA. Therefore, the noninvasive diagnosis of CHC is still very important. In general, the performance of this model in distinguishing CHC from HCC was similar to that in LI-RADS v2017, which may be helpful in clinical practice for CHC inpatients.

There are limitations in our study. First, the sample size of CHC participants in our study was not large. Second, our data were from a single center, and the results need to be expanded to other centers to confirm its reproducibility. Third, US is operator-dependent and has lower sensitivity in overweight and obese patients, and all potential nodules may not be found with cross-sectional imaging by US. Finally, there is the possibility of overfitting during the model development.

### Conclusions

In conclusion, we developed an ultrasomics model for the preoperative differentiation of CHC from HCC, which showed higher ability than CE US LI-RADS v2017 for differentiating CHC from HCC though the difference was not significantly different. This model may be helpful to differentiate CHC from HCC in clinical diagnosis.

## Supplementary Information

**Additional file 1.** Development of U model and the important features.

## Data Availability

The datasets used and analyzed during the current study are available from the corresponding author on reasonable request.

## Abbreviations

CHC: Combined hepatocellular cholangiocarcinoma
HCC: Hepatocellular carcinoma
CCA: Cholangiocarcinoma
CE: Contrast enhanced
LI-RADS: Liver imaging reporting and data system
CA19-9: Carbohydrate antigen 19-9
AFP: Alpha-fetoprotein
TACE: Transarterial chemoembolization
FLL: Focal liver lesions
ROI: Region of interest
U-score: Ultrasomics score
U model: Ultrasomics model
ROC: Receiver operating curve
AUC: The area under the curve
CI: Confidence interval
TIV: Tumor in vein
PPV: Positive predictive value
NPV: Negative predictive value
SVM-RFE: Support vector machine recursive feature elimination
SVM: Support vector machine
