# Correction to: Differentiation between combined hepatocellular cholangiocarcinoma and hepatocellular carcinoma: comparison of diagnostic performance between ultrasomics-based model and CEUS LI-RADS v2017

**DOI:** 10.1186/s12880-022-00781-x

**Published:** 2022-03-29

**Authors:** Chao-qun Li, Xin Zheng, Huan-ling Guo, Mei-qing Cheng, Yang Huang, Xiao-yan Xie, Ming-de Lu, Ming Kuang, Wei Wang, Li-da Chen

**Affiliations:** 1 Department of Medical Ultrasonics, Institute of Diagnostic and Interventional Ultrasound, The First Affiliated Hospital of Sun Yat-Sen University, 58 Zhongshan Road 2, Guangzhou, 510080 People’s Republic of China; 2 Department of Hepatobiliary Surgery, The First Affiliated Hospital of Sun Yat-Sen University, Guangzhou, China

## Correction to: BMC Medical Imaging (2022) 22:36 https://doi.org/10.1186/s12880-022-00765-x

Following the publication of the original article [1], the authors requested to amend the article title from “Differentiation between combined hepatocellular carcinoma and hepatocellular carcinoma: comparison of diagnostic performance between ultrasomics-based model and CEUS LI-RADS v2017” to “Differentiation between combined hepatocellular cholangiocarcinoma and hepatocellular carcinoma: comparison of diagnostic performance between ultrasomics-based model and CEUS LI-RADS v2017”.

The correct title is included in this Correction and has already been updated in the original article.
