# Supplementary material for: Differentiation between combined hepatocellular cholangiocarcinoma and hepatocellular carcinoma: comparison of diagnostic performance between ultrasomics-based model and CEUS LI-RADS v2017
Source: BMC Med Imaging. 2022 Mar 3;22:36. doi: 10.1186/s12880-022-00765-x (PMC8896152; doi:10.1186/s12880-022-00765-x)
Supplement: Supplementary file 1 — Additional file 1. Development of U model and the important features. [file 12880_2022_765_MOESM1_ESM.docx]

**supplementary materials**

*Ultrasomics features extraction and ultrasomics models*

There are 5936 features for selection in the Ultrasomics-Platform after loading data, including Original, Ipris, CoLIAGe, Wavelets+LBP, Shearlets, GLBP, GLDP etc. We selected all features and then performed automatic feature extraction. 5936 features were extracted from one single ROI, a total of 23744 features from each patient with four images. After loading the data of extracted features, the ratio of splitting training set and validation set and the methods of missing value processing, oversampling and data normalization are selected in the preprocessing part of Ultrasomics-Platform. We selected the ratio of training set was 0.8, and median filling missing value, none oversampling, z-score standardization in this study.

In feature selection and machine learning module, Ultrasomics-Platform provided 5 methods for feature selection including Spearman, Statistical Test, SVM-RFE, L1 regularized Logistic Regression, Random Forest, and 10 machine learning algorithms including Decision Tree, Naïve Bayes, KNN, Logistics Regression, SVM, Bagging, Random Forest, Extremely Randomized Trees, Adaboost and Gradient Boosting Tree.

This module will automatically model by various combinations of feature selection methods and machine learning algorithms, and display the ROC curve of each model. Optimal model with highest AUC was used as the final model. Our model was developed based on spearman+SVM-RFE+SVM. High correlation features is removed with 0.75 as the threshold by spearman rank correlation analysis, then using support vector machine-recursive feature elimination for feature selection. Finally, the selected features were used to build the model by SVM, and Ultrasomics-Platform automatically tuning of hyperparameters of SVM for optimal parameters by GridSearchCV.

*Important features of the U model*

We selected important features in U model including :

shearlet2DIdxs[2 3 1]_gldm_DependenceVariance,

shearlet2DIdxs[1 3 4]_gldm_HighGrayLevelEmphasis,

shearlet2DIdxs[ 1 2 -1]_glrlm_ShortRunEmphasis,

shearlet2DIdxs[ 1 3 -4]_glcm_Autocorrelation,

shearlet2DIdxs[ 1 2 -2]_glrlm_LowGrayLevelRunEmphasis,

shearlet2DIdxs[1 3 0]_glszm_SmallAreaHighGrayLevelEmphasis,

shearlet2DIdxs[2 2 1]_firstorder_Skewness,

shearlet2DIdxs[1 3 1]_glrlm_GrayLevelVariance,

shearlet2DIdxs[ 1 3 -1]_firstorder_Entropy,

shearlet2DIdxs[2 1 0]_glcm_Idmn,

CoLIAGe2D_WindowSize11_Entropy_firstorder_90Percentile,

CoLIAGe2D_WindowSize5_Sum Variance_firstorder_Maximum,

CoLIAGe2D_WindowSize3_Sum Entropy_firstorder_Median,

CoLIAGe2D_WindowSize3_Sum Average_firstorder_Range,

wavelet-HLL_lbp-3D-m2_firstorder_10Percentile,

wavelet-HHL_lbp-3D-k_firstorder_Minimum,

gldp_hist_0_kernel2_8,

gldp_hist_90_kernel5_4,

gldp_hist_45_kernel0_6.
